# Balloon aortic valvuloplasty as a palliative treatment in patients with severe aortic stenosis and limited life expectancy: a single center experience

**DOI:** 10.18632/aging.103862

**Published:** 2020-08-27

**Authors:** Francesca Mantovani, Marie-Annick Clavel, Antonella Potenza, Chiara Leuzzi, Teresa Grimaldi, Luigi Vignali, Alessandro Navazio, Vincenzo Guiducci

**Affiliations:** 1 Azienda Unità Sanitaria Locale - IRCCS di Reggio Emilia, Cardiology, Reggio Emilia, Italy; 2 Institut Universitaire de Cardiologie et de Pneumologie, Québec, Canada; 3 Division of Cardiology, Azienda Ospedaliero Universitaria di Parma, Parma, Italy

**Keywords:** aortic stenosis, balloon aortic valvuloplasty, heart failure, limited life expectancy, palliation

## Abstract

Whether balloon aortic valvuloplasty (BAV) may provide an effective palliation in symptomatic high-risk patients is uncertain. Therefore, we aimed to evaluate outcomes in symptomatic high-risk patients with severe aortic stenosis (AS), who underwent BAV. All-cause mortality and length of hospitalization for heart failure (HF) up to death or to 1-year follow up were collected after BAV. One hundred thirty-two (132) patients (62% women), mean age 85±7 years, underwent BAV with a substantial reduction of the peak-to-peak aortic gradient from 53±21 to 29±15 mmHg (p<0.001). The median of days of HF hospitalization prior to BAV was 9 (0-19), and decreased after BAV to 0 (0-9), p<0.001. During 1-year follow-up patients with untreated CAD (85, 64%) had a higher mortality compared to patients with insignificant/treated CAD (47, 36%): 1-year survival: 45±7% vs. 66± 7%; p=0.02. After adjustment for STS risk score and severity of residual AS, patients with untreated CAD remained at higher risk of mortality (adjusted HR 1.74 [1.01-2.91]; p=0.04). Thus, in this series of symptomatic high-risk patients, BAV was associated with a significant reduction in aortic valve gradient and hospitalization time for HF post-BAV. In patients with significant CAD, percutaneous intervention might be considered in order to improve survival.

## INTRODUCTION

After its introduction, more than 30 years ago [1], as a possible treatment for severe aortic stenosis (AS), balloon aortic valvuloplasty (BAV) was considered unacceptable due to perceived procedural complexity, high restenosis rates and mortality rates post-BAV similar to those of untreated AS [2–5]. The resurgence of BAV in the last decade is related to its use as a prelude to transcatheter aortic valve implantation (TAVI). Subsequently, BAV technique and technology improved and operators acquired experience which has lead to improved safety [6–8].

Despite current guidelines considering BAV only as a bridge to surgical aortic valve replacement (SAVR) or TAVI [9, 10], BAV may provide in daily practice a palliative treatment option for patients with significantly reduced life expectancy (malignancy, dementia, liver disease, etc.) for whom no other invasive therapy (TAVI nor SAVR) is indicated [9]. These difficult-to-treat patients, together with the frail and very elderly, represent a substantial part of the AS population. This part will be even broader over the next decade due to the ageing of the general population [11]. However, the standardization and framework of BAV use as a palliative treatment are not provided so far. Moreover, frequent coexistence of coronary artery disease (CAD) and AS [12] makes the clinical management even more difficult, raising the question of revascularization impact in this specific subset of patients.

Therefore, the aims of the study were to: 1) evaluate procedural success and early event-rate of BAV, 2) evaluate 1-year mortality and hospitalization for heart failure (HF) in patients with severe AS undergoing BAV, and 3) evaluate the impact of CAD on 1 year-mortality in this high-risk population post-BAV.

## RESULTS

### Study population

The baseline demographic and clinical characteristics of the study population are presented in the Table 1. Among our 132 patients, 62% were women, mean age was 85±7 years with 19% patients older than 90 years old. Most patients (85%) were symptomatic for HF with a New York Heart Association (NYHA) functional class III-IV. Syncope was reported in 16% of cases and angina in 19%. The estimated procedural risk calculated via the STS score was 6±4%.

**Table 1:** Baseline characteristics of the population (left column) and comparison between patients with untreated vs. insignificant/treated coronary artery disease (CAD).

	**Whole cohort n=132**	**Insignificant/treated CAD n=85; 64%**	**Untreated CAD n=47; 36%**	**p**
Female gender, n (%)	82 (62%)	61 (72%)	21 (45%)	**0.002**
Age, years	85±7	85±7	84±6	0.22
Age ≥90 years, n (%)	25 (19%)	21 (25%)	4 (9%)	**0.02**
Body mass index, kg/m^2^	26±4	25±4	26±3	0.76
Body weight <50kg	13 (10%)	9 (11%)	4 (9%)	0.69
**Comorbidities**:				
Smokers, n (%)	22 (17%)	13 (15%)	9 (19%)	0.57
Diabetes, n (%)	26 (20%)	10 (12%)	16 (34%)	**0.002**
Previous known CAD, n (%)	37 (28%)	21 (25%)	16 (34%)	0.25
Significant CAD before BAV, n (%)	72 (55%)	25 (29%)	47 (100%)	
Number vessels diseased (among patients with significant CAD before BAV):		0.32
1 vessel diseased:	34 (48%)	12 (48%)	22 (47%)	
2 vessels diseased:	16 (23%)	8 (32%)	8 (19%)	
3 vessels diseased:	21 (30%)	5 (20%)	16 (34%)	
Left main involvement:	11 (15%)	3 (12%)	8 (17%)	0.57
Creatinine, mg/dl	1.3 (1.0-1.8)	1.3 (1.0-1.8)	1.5 (1.1-2.1)	0.22
Chronic kidney disease (≥moderate)	25 (18%)	15 (18%)	9 (19%)	0.83
Haemoglobin, g/dl	10.7 (9.2-12.3)	10.6 (8.9-12)	10.9 (9.5-13.3)	0.19
STS score	6±4%	6±4%	7±4%	0.74
Dementia, n (%)	27 (20%)	16 (19%)	11 (23%)	0.53
Peripheral artery disease, n (%)	53 (40%)	24 (28%)	29 (62%)	**0.0002**
CVA/TIA	24 (18%)	15 (18%)	9 (19%)	0.83
Chronic obstructive pulmonary disease, n (%)	32 (24%)	19 (22%)	13 (24%)	0.50
Localized solid cancer, n (%)	37 (28%)	24 (28%)	13 (28%)	0.94
Metastatic cancer, n (%)	4 (3%)	2 (3%)	3 (4%)	0.54
Physical disability, n (%)	26 (20%)	20 (24%)	6 (13%)	0.14
**Symptoms at presentation**				
NYHA class III-IV, n (%)	112 (85%)	69 (81%)	43 (91%)	0.11
Syncope, n (%)	21 (16%)	14 (16%)	7 (15%)	0.81
Angina at presentation, n (%)	25 (19%)	15 (18%)	10 (21%)	0.61
**Echocardiographic characteristics**:				
Left Ventricle Ejection Fraction, %	49±13	50±12	48±15	0.24

A substantial burden of comorbidities was observed: known CAD was present in 28% of the patients, dementia in 20%, chronic kidney disease (at least moderate) in 18%, physical disability in 20%, history of localized solid cancer in 28% and metastatic cancer in 3%. The body weight was below 50 kg in 10% of patients. Left ventricular ejection fraction was severely reduced (<35%) in 23% of patients and significant mitral regurgitation (at least moderate) was present in 39% of patients. Taken together all these characteristics contributed to an important frailty of the population, justifying the Heart Team’s decision regarding the exclusion of any invasive therapy, except palliative BAV.

### Procedural data

**Figure 1:**
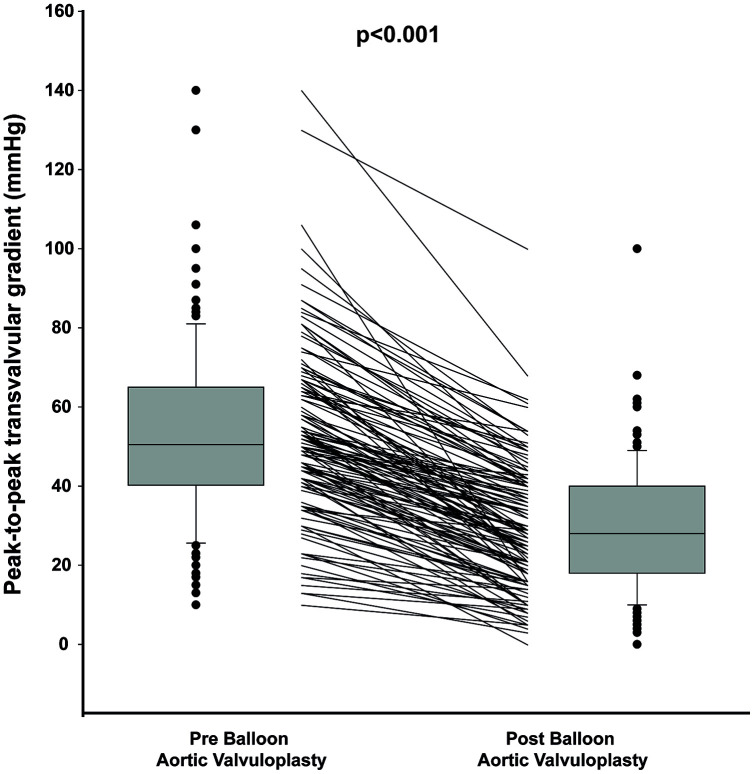
**Peak to peak gradient pre and post Balloon Valvuloplasty.** The figure shows the median, percentile 25 and 75 as well as individual values of peak-to-peak gradient pre and post valvuloplasty. The box plots of the distribution show the median, percentile 25 and 75, the whiskers (1.5 times the interquartile range) and the outliers.

BAV induced a substantial reduction of the peak-to-peak aortic valve gradient from 53±21 to 29±15mmHg (p<0.001; Figure 1), with a decrease in gradient of at least 50% in 65% of the patients. A worsening in aortic regurgitation after BAV was observed only in 2 patients (in both cases from mild to moderate aortic regurgitation).

All the procedures were performed with a low rate of complications (Table 2). Three patients (2.3%) had a vascular complication following femoral access for BAV (online supplement). Only one patient, who underwent BAV in cardiogenic shock, died intraoperatively. One patient had a stroke a few hours after the procedure. No cardiac tamponade, acute limb ischemia, need for permanent pacemaker implantation was observed. All causes mortality at 30 days was 5%.

**Table 2:** Procedural data.

	**Whole cohort n=132**		**No/treated CAD n=85; 64%**	**Untreated CAD n=47; 36%**	**p**
*Peak to peak gradient pre, mmHg*	50.5 (40.3-65.0)		52.0 (41.0-68.5)	49.0 (35.0-55.0)	0.06
*Peak to peak gradient post, mmHg*	28.0 (18.0-40.0)		30.0 (21.0-41.8)	24.0 (11.0-36.0)	**0.01**
*Delta gradient*	20.0 (14.0-31.0)		20.0 (14.0-30.8)	22.0 (14.0-32.0)	0.83
*Percentage of gradient decrease post-BAV>=50%*	85 (65%)		59 (70%)	25 (55%)	0.08
*Vascular complications, n (%)*	3 (2.3%)		2 (2.4%)	1 (2.1%)	0.93

Repeated BAV was necessary in 8 patients (6%) during 1-year follow-up (online supplement).

### Mortality and hospitalization for heart failure during 1-year follow-up

**Figure 2:**
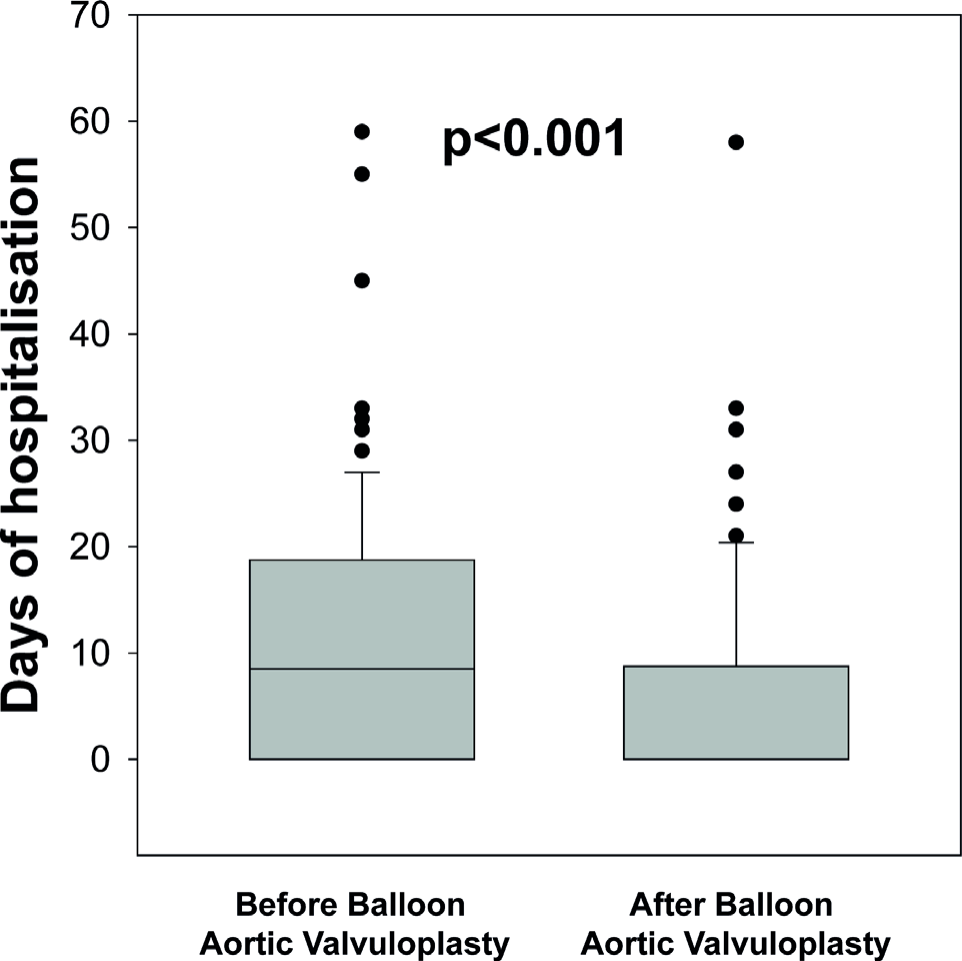
**Comparison of days of heart failure hospitalization before and after balloon aortic valvuloplasty (BAV) in patients with no surgical or transcatheter therapeutic option for symptomatic severe aortic stenosis.** The box plots show the median, percentile 25 and 75, the whiskers (1.5 times the interquartile range) and the outliers.

During 1-year follow-up, 57 patients (43%) died. In the whole cohort, the median of days of HF hospitalization prior BAV was 9 (0-19) and decreased after BAV to 0 (0-9), p<0.001 (Figure 2). Therefore, the time spent in hospital has been more than halved, from 8.1%±19.7% prior BAV to 2.7%±5.6 after BAV (p<0.001). The median of days before discharge after BAV was 3 [2–5].

In the subgroup of patients (n=89, 67%) that had HF hospitalizations prior to BAV [median of days of hospitalization 13 (8–13)], the number of days of hospitalization was decreased to 0 (0-14) after BAV, p<0.001, and 48 patients (54%) were not hospitalized after BAV. In 48 patients without HF hospitalization before BAV, only 9 (21%) had HF hospitalizations after BAV.

Interestingly, the number of days of HF hospitalization in the entire year before BAV (in all patients: 12(5–25) days) was an independent predictor of mortality (HR: 4.51[1.38-13.61]; p=0.001). The best threshold to predict increased mortality post-BAV was 12 days of HF hospitalization in the year before BAV, with a sensibility of 40% and a specificity of 83%. Patients with ≥12 days of HF hospitalization in the year before BAV had more than a 2-fold increase in mortality post-BAV (HR: 2.32[1.38-3.94]; p=0.002) which remained significant after adjustment for STS score, post BAV peak-to-peak gradient and untreated CAD (HR: 1.95[1.14-3.33]; p=0.01).

### Coronary artery disease

Thirty-seven (28%) patients had known coronary artery disease (CAD) in previous history. Prior myocardial infarction was reported in 24 patients (18%), and previous coronary artery bypass grafting in 10 patients (8%). During the index hospitalization for BAV 60 patients (45%) were found with no significant CAD. Of the 72 patients (55%) that were found with significant CAD, 34 (26%) had a single vessel disease, 17 (13%) had a 2-vessel disease and 21 (16%) had a 3-vessel disease; in 11 patients (8%) left main artery was involved. Of these patients found with significant CAD: 13 (10%) underwent complete revascularization, 12 (9%) underwent incomplete revascularization and 47 (36%) were left untreated during the index hospitalization. Therefore, the main cohort was divided in two groups: 85 patients (64%) with insignificant/treated CAD and 47 (36%) patients with untreated CAD. Comparison between groups (Table 1) showed in the untreated CAD group a higher prevalence of male gender, diabetes and peripheral artery disease compared to the insignificant/treated CAD group (all p>0.002). STS score as well as the reported symptoms related to AS (heart failure symptoms as NYHA class III-IV, angina, syncope/pre-syncope) were similar in the two groups (all p>0.1). Peak-to-peak aortic gradient pre-BAV was equivalent in the two groups, however a lower peak-to-peak aortic gradient post BAV was obtained in the untreated CAD group (p=0.03). Surprisingly, among

patients with CAD, those left untreated did not have larger extent/complexity of CAD (Table 1).

The median of days of discharge time after BAV showed no significant difference between the two groups (median: 3 days [2–5] in the insignificant/treated CAD group and 4 days [2–6] in the untreated CAD group, p=0.4). Occurrence of vascular complications, intraprocedural death and 30-days mortality were similar in the two groups (all p>0.22).

**Figure 3:**
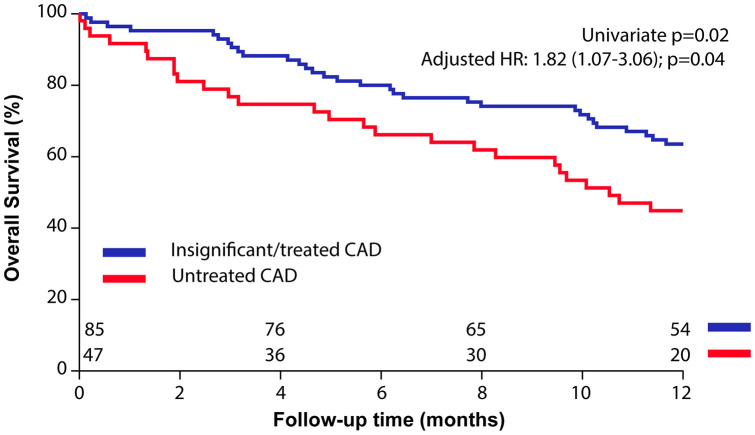
**Impact of untreated coronary artery disease (CAD) in patients who underwent balloon aortic valvuloplasty (BAV) for symptomatic severe aortic stenosis.** Kaplan-Meier curves of overall survival after BAV in patients with untreated vs. insignificant/treated CAD.

During 1-year follow-up patients with untreated CAD had a higher mortality compared to patients with insignificant/treated CAD (Survival: 6-month: 64±5% vs. 80±4%; 1-year: 45±7% vs. 66± 7%; p=0.02; HR: 1.82 (1.07-3.06); p=0.02, Figure 3).

After adjustment for STS risk score and severity of residual AS (as documented by peak-to-peak gradient post-BAV), patients with untreated CAD remained at higher risk for mortality (adjusted HR 1.74 [1.01-2.91]; p=0.04).

When CAD was analysed in 3 groups (i.e. isolating incomplete revascularization), patients with untreated CAD remained at higher risk of mortality (HR: 1.73[1.08-3.03]; p=0.04) compared to patients with insignificant or completely revascularized CAD (cf. online supplement).

## DISCUSSION

Our study shows that, in the elderly population with severe symptomatic AS and limited life expectancy due to advanced age, comorbidities and frailty (thus not suitable for definitive treatment according to the local Heart Team): 1) BAV has a low rate of procedural complications and low short-term overall mortality, so it may be considered safe, 2) single (or repeated) BAV may improve the patients quality of life since it halves the time spent in hospital for heart failure decompensation, 3) the concomitant presence of significant CAD at the time of BAV, if left untreated, may increase the 1-year overall mortality. The present study is, to our knowledge, unique in literature so far.

### BAV and the value of symptoms’ palliation in patients with limited life expectancy

The Euro Heart Survey highlighted that almost one third of patients were considered too high risk for surgery because of significant comorbidity. These included advanced age, severe LV dysfunction, chronic obstructive pulmonary disease, previous stroke, and impaired renal function. [13] In this subset of patients deemed too high risk for SAVR, TAVI has become an established alternative treatment [14] providing reduction in mortality and symptomatic improvement when compared to conservative therapy [15]. As the use of TAVI increases, primary care physicians and general cardiologists may be more inclined to refer frail elders with end-stage AS instead of managed them medically. As reported in the current guidelines, TAVI is not recommended in patients in whom existing comorbidities would preclude the expected benefit from correction of AS [10]. The increasing number of poor candidates for both SAVR and TAVI in the expanding very elderly population mandates alternative methods such as BAV to alleviate AS symptoms burden and improve quality of life. Therefore, patients who require BAV as palliative strategy represent the highest end of the spectrum risk of those with severe symptomatic AS. The proposed advantages of BAV in this subset of patients include a short-term survival benefit of BAV over medical therapy at 3 and 6 months [16, 17]. Also, quality of life in BAV patients compare very favorably with conservatively managed patients at 6 months while the benefit of BAV as a standalone procedure has been reported to be lost within 12 months follow up [17].

The value of symptoms’ palliation in the population in which life-expectancy is limited, as consequence of frailty, advanced age, and multiple comorbidities, cannot be understated. It should be accepted that BAV is a temporary treatment, that, nevertheless, minimize the need for repeated hospitalizations, as our data showed. BAV has thus an important impact on quality of life for these elders over 80 and 90 year old, and may be potentially cost saving. In our study 8% of patients underwent repeated BAV, this highlights the fact that in order to achieve symptoms relief and reduction in the HF hospitalization rates, patients need to be followed up regularly to monitor for evidence of clinically significant restenosis.

Despite the risk profile of the study population, the complication rate remained low, and compared very favorably with other reported series. [5, 6, 16–18] Of note, those studies reported in turn an improvement in both procedural and in-hospital mortality [6, 19] when compared to earlier studies [1, 19]. Our encouraging outcomes reflect improved equipment and technology, including the use of rapid right ventricular pacing during the procedure, which was not employed in early series. We reported no aortic rupture or worsening of aortic regurgitation to the highest grade, likely a reflection of a conservative balloon size, as well as the selection of the patients. Also, the low access site complications in this current report was favorable and probably related to the small sheath size (10 French) and the use of vascular closure devices. The 1-year mortality rate was 43% in our study which is in line with other old or recent series, the improvement of the procedure probably counterbalanced by a worse health status of the patients. In 1994, Otto et al. [3] reported a 45% in 1-year mortality after undergoing successful BAV for symptom palliation.

In recent studies, patients managed medically had a survival rate at 1-year comprised between 40 and 60%, with or without BAV [17, 20–22]. Thus, BAV as a definitive therapy does not seem to change the natural history of the disease and the dreadful outcome that untreated severe AS achieves: in 453 patients who did not undergo SAVR between 1993 and 2003 the overall mortality was 38% at 1 year [23].

### The CAD dilemma: To treat or not to treat concomitant significant CAD in the setting of severe aortic stenosis undergoing BAV

According to the current guidelines [9, 10], patients with severe AS and concomitant CAD should preferably be treated with coronary artery bypass grafting concomitant to SAVR. However, PCI is considered in patients with significant stenosis of major epicardial vessels not suitable for surgery and undergoing TAVI. [9] The available evidence in support of different revascularization strategies is mostly based on retrospective, single-center studies reporting unadjusted and discordant outcomes [24–29]. The ongoing PCI prior to TAVI (ACTIVATION) study is the first randomized controlled trial designed to evaluate non-inferiority of PCI compared with not treating such coronary lesions before TAVI [30]. In the setting of AS and significant CAD in patients with an indication for palliative BAV, data are even more scarce, raising many doubts on the management of these patients. In theory, BAV with PCI may also be a admissible treatment option in this group of patients without ideal AS treatment option (SAVR/TAVI).

As in our reports, the latest and largest registry of 2127 procedures from hospitals in the United States comparing BAV versus BAV with PCI has reported no difference in in-hospital mortality, length of hospital stay, and procedural complications [31]. The difference in 1-year mortality between patients with significant CAD left untreated and patients with insignificant/treated CAD (even if it was not entirely treated) is of notice and in line with the only previous study comparing BAV with BAV and PCI [32]. Despite the International Study of Comparative Health Effectiveness with Medical and Invasive Approaches (ISCHEMIA) [33] did not show evidence that an initial invasive strategy versus an initial conservative strategy reduced the risk of ischemic cardiovascular events or death from any cause among patients with stable coronary disease and moderate or severe ischemia, these results cannot be extended to our population given the relatively low risk of the trial patients.

Our study results suggest that addressing at least the main coronary lesions at the time of BAV, might lead to survival benefits without increased risks at the time of procedure. While awaiting further data to provide more solid evidences, the decision to pursue coronary revascularization in high-risk BAV patients could be tailored by the Heart Team on case-by-case basis.

### Limitation

Our study has the intrinsic limitations of an observational retrospective, single center study, therefore some degree of bias cannot be excluded. Despite its limitations, this ‘real-world’ and ageing population treated by contemporary BAV practice supports the clinical utility of BAV. Moreover, this study is unique in literature since only the palliative indication for BAV was considered. Indeed, as all studies reported results from various BAV indications (palliation, bridge to SAVR or TAVI and diagnostic), thus with heterogenous cohort’ characteristics, especially in terms of patients’ risk profile, and therefore difficult to apply to clinical practice.

Selection of patients to undergo PCI as well as BAV was based on the clinical judgment of the operators and the local Heart Team. Thus, unmeasured factors may have influenced BAV outcomes. CAD is a heterogeneous and complex disease, especially in terms of extent of injured myocardium and lesion complexity, that we were not able to fully assess in our study. Indeed, the Heart Team could have decided to not perform revascularization in patients with chronic and complex coronary artery disease, while in patients with non-complex and single isolated artery stenosis revascularization could have been favored, despite the similar degree/extend of CAD we reported. For this purpose, stratifying patients according to disease severity – i.e. by means of Syntax score – may have allowed assessment of the prognostic implications of CAD on clinical outcomes after BAV with greater accuracy. Therefore, further studies specifically on concomitant use of BAV and PCI are needed to validate our observations.

## CONCLUSION

In this series of high risk, elderly, frail and comorbid patients with symptomatic severe aortic stenosis, BAV as a palliative procedure was performed safely with a low complication rate and low procedural mortality. Moreover, BAV was associated with a significant reduction in aortic valve gradient and it decreased the hospitalization time for heart failure post BAV. This supports an important role for BAV in palliation of symptoms in patients with limited life expectancy in whom the role of TAVI is uncertain or inappropriate. Finally, in this subset of old and multi-comorbid patients, CAD might be detrimental if left untreated, while addressing at least the main coronary lesions does not seem to carry the same burden.

## MATERIALS AND METHODS

A retrospective analysis of 10 years of practice of BAV as a palliative strategy in patients with symptomatic AS between March 2008 and June 2018 at Arcispedale Santa Maria Nuova, Reggio Emilia, Italy, was performed. Demographic, clinical, procedural, and 1-year follow-up data on all patients were collected.

Indication for BAV was established by the local Heart Team, consisting in patients not suitable to SAVR or TAVI because of a limited life expectancy (<1 year), in which BAV was intended as palliative and final strategy. Patients who received BAV as a bridge to SAVR or TAVI, and residents outside Reggio Emilia province were excluded. Patients were followed-up for 12 months.

Informed consent for the BAV procedure was obtained from each patient. The study protocol conforms to the ethical guidelines of the 1975 Declaration of Helsinki and the study was approved by the Institutional Review Board who waived patient’s individual consent due to the retrospective nature of the study.

### Clinical assessment

Preprocedural demographic and clinical data were collected during systematic consultation of patients’ charts. The surgical risk was estimated by the Society of Thoracic Surgeons Predicted Risk of Mortality (STS) score (http://riskcalc.sts.org/stswebriskcalc/calculate).

Severe AS was defined by a mean transvalvular gradient >40 mmHg, and/or aortic valve area <1.0 cm^2^ (indexed aortic valve area <0.6 cm^2^/m^2^) at echocardiography [34].

Life expectancy of less than 1 year was determined according to best attempted clinical estimate.

### Coronary artery disease

Significant CAD was defined by invasive coronary angiography as >50% stenosis of the left main, or >70% stenosis in ≥1 major coronary vessel, (left anterior descending artery, left circumflex artery, and right artery). In presence of significant CAD, the Heart Team decided case by case if: proceed with a complete revascularization of the lesions, address only the main lesion (incomplete revascularization), or leave the CAD untreated.

The main cohort was divided in two group: a group with non-significant CAD or CAD that was completely or incompletely percutaneously treated during the index hospitalization for BAV and a group with CAD left untreated during the index hospitalization for BAV.

### Procedures

All the procedures were performed according to standard techniques via the retrograde femoral approach (complete description in the online supplement). In case of severe coronary stenosis of primary vessels, percutaneous coronary intervention (PCI) was usually immediately performed and BAV organized in another session. During BAV, the peak-to-peak gradients was recorded using pig-tail catheter in the left ventricle and the side arm of the sheath for the aortic pressure. The procedure was considered successful if the pre-procedural peak-to-peak gradient decreased by at least 50% after procedure. An aortogram was performed to assess aortic regurgitation only if diastolic pressures were considered abnormal.

### Endpoints

The primary endpoint of the study was the change in the number of days of hospitalization for HF prior to and post-BAV.

The secondary endpoints were the procedural success, the early event rate and the overall 1-year mortality according to the significance/treatment of CAD.

### Follow-up and events’ collection

**Figure 4:**
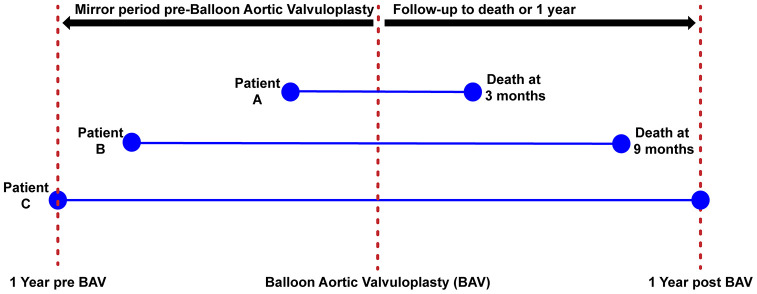
**Mirror period for the evaluation of days of heart failure hospitalization.** The figure is describing the method to assess the number of days of hospitalization before/after BAV. Patients 1 and 2 died before 1-year post BAV, thus the time period used to record the number of days of HF hospitalization was less than 1 year in these patients. Patient 3 survived more than 1 year, thus the entire year before BAV was used to collect number of days of HF hospitalization.

We collected the incidence and number of days of hospitalization for HF post BAV up to 1-year follow-up (or death) and prior BAV in a mirrored period of interrogation. Thus, pre-BAV, the interrogation period finished one day and started one year before BAV (i.e. the days of HF hospitalization before BAV, from admission to index procedure, if any, were included). However, if a patient died before one year of follow-up, the interrogation period in which we count days of HF hospitalisation before BAV was reduced to the exact same number of days lived by the patient after BAV (Figure 4).

The number of days of hospitalisation as well as the diagnosis of HF were derived from hospital discharge codes using the electronic archives of the health service of Reggio Emilia province.

For secondary endpoints, the event collected were procedural success, early complication, all-cause mortality at 30 days and at 1 year after BAV, and the percentage of patients having repeated BAV at 1-year follow-up. Follow-up information for death was obtained from the national death index, where the status of all citizens is steadily updated. Thus, follow-up for all endpoints was 100% complete.

### Statistical analysis

Data are presented as percentages for categorical variables and as mean ±SD or median (percentile 25-75) for continuous variables according to the type of distribution. Normal distribution was tested with the use of Shapiro-Wilk test. The change in number of days of HF hospitalization before/after BAV was analysed by a Wilcoxon signed-rank test. Direct comparison between patients with or without significant CAD at discharge used t-tests or Wilcoxon tests and chi-square or Fischer exact tests as appropriate. Survival and HF rates, estimated using Kaplan-Meier method, were compared using log-rank test. Cox-proportional-hazards analyses were used to estimate the relative risk of death and are presented as hazard-ratios (HR) with 95% confidence intervals. Multivariable Cox analysis were adjusted for STS risk score (as opposed to individual variables to ascertain adequate statistical power) and severity of residual AS after BAV (i.e. peak to peak gradient).

All tests were two tailed. A P<0.05 was considered statistically significant. Analyses were performed using JMP version 9.0.1 (SAS Institute Inc., Cary, NC).

## Supplementary Material

Supplementary Materials
