# Supplementary material for: Balloon aortic valvuloplasty as a palliative treatment in patients with severe aortic stenosis and limited life expectancy: a single center experience
Source: Aging (Albany NY). 2020 Aug 27;12(16):16597–608. doi: 10.18632/aging.103862 (PMC7485726; doi:10.18632/aging.103862)
Supplement: Supplementary Materials [file aging-12-103862-s001..pdf]

SUPPLEMENTARY MATERIALS

Procedures description

All the procedures were performed according to standard techniques via the retrograde femoral approach.

During the PCI patients received 70 U/Kg unfractionated heparin in order to achieve an activated clotting time of 200–300 sec. Angiographic success was defined as a residual coronary stenosis inferior to 30% with Thrombolysis in myocardial infarction flow grade 3.

BAV was carried out with a Perclose or Proglide pre-implantation and a 10F femoral sheath insertion. Unfractionated heparin was administered in all patients at a dose of 10-50 UI/kg. In order to stabilize the balloon during inflation the right ventricle was paced at180-200 bpm.

All valvuloplasty were performed with Cristal Balloon (AB Medica, from 18 mm to 23 mm diameter). Two consecutive balloon inflations were performed before measuring the post dilatation gradient. Arterial puncture sites were closed by using 6F Proglide/Perclose.

Potential reason for denying SAVR or TAVI

Among our 132 patients, 32 patients were referred for BAV due to high surgical risk (STS score >8%), 17 for age >90 years old, 39 for age>85 years old and low BMI, 4 for end stage renal failure and 40 the decision remained on the heart team and was not specified.

Vascular complications following BAV

One patient had a large hematoma successfully managed with manual bandage compression with no further consequences.

One patient had a dissection of the right common iliac artery with a small hematoma formation and mild anemization.

One patient had a dissection and thrombosis of the right deep femoral artery requiring urgent thromboendarterectomy and percutaneous transluminal angioplasty and need for blood transfusion.

Patients who required a second BAV within 1-year follow-up

Patients who underwent a second BAV did not present any baseline specificity.

	**Patients with second BAV within 1 year (n=8)**
Age, years	85.6 ±2.6
Female gender, n (%)	5 (62%)
Body Mass Index, kg/m^2	25.8 ±3.7
STS score	6.5 ±1.8
Creatinine, mg/dl	1.3 (1.1-2.2)
Haemoglobin, g/dl	10.7 (9.7-13.0)
NYHA class III-IV, n (%)	8 (100%)
Syncope, n (%)	1 (12.5%)
Angina at presentation, n (%)	0 (0%)
Ejection fraction (%)	48 (33-60)
Peak-to-peak gradient pre BAV, mmHg	45.0 (33.5-66.3)
Peak-to-peak gradient post BAV, mmHg	23.5 (15-37.8)
Delta gradient, mmHg	17.5 (12.0-35.8)

The characteristics of the second BAV were:

**Patient**	**Peak to peak gradient pre second BAV, mmHg**	**Peak to peak gradient post second BAV, mmHg**	**Number of days between index BAV and repeated BAV**
1	50	39	294
2	65	22	194
3	38	25	275
4	23	13	200
5	39	28	241
6	55	39	342
7	20	12	143
8	60	28	283

Analysis of CAD treatment

CAD was divided in 3 different groups:

- Patients with insignificant or completely revascularized CAD (n=73; 55%),
- Patients with incomplete revascularization (n=12; 9%), and
- Patients with untreated CAD (n=47; 36%).

After adjustment, patients with untreated CAD had higher mortality than patient with insignificant or completely revascularized CAD (HR: 1.73[1.08-3.03]; p=0.04). Interestingly, there was also a trend toward higher mortality in patients with untreated CAD compared to patients with incomplete revascularization (HR: 1.79[0.90-7.28]; p=0.08). However, there was not difference in mortality between patients with insignificant or completely revascularized CAD and patients with incomplete revascularization (HR: 0.96 (0.22-1.88), p=0.52).

These results need to be taken with caution given the small number of patients.
